# MicroRNA-455-3p promotes invasion and migration in triple negative breast cancer by targeting tumor suppressor EI24

**DOI:** 10.18632/oncotarget.14307

**Published:** 2016-12-27

**Authors:** Zhishuang Li, Qingyong Meng, Aifeng Pan, Xiaojuan Wu, Jingjing Cui, Yan Wang, Li Li

**Affiliations:** ^1^ Department of Pathology, Shandong University, School of Medicine, Jinan, Shandong, 250012, P.R. China; ^2^ The No. 2 People's Hospital of Jinan, Jinan, Shandong, 250001, P.R. China; ^3^ Department of Thoracic Surgery, Shandong University, Qilu Hospital, Jinan, Shandong, 250012, P.R. China

**Keywords:** miR455-3p, triple negative breast cancer, invasion, migration, EI24

## Abstract

Lacking of treatment methods for the patients with triple negative breast cancer (TNBC) underscores the pivotal needs to further understand its biology as well as to find better biomarkers and develop novel therapeutic strategies. Increasing evidences support that aberrantly expressed microRNAs (miRNAs) are involved in tumorigenesis and may serve as biomarkers for diagnostic and prognostic purposes of various cancers. In current study, we found that miR-455-3p and miR-196a-5p were intensively overexpressed in TNBC compared with the hormone receptor (HR) positive breast cancer whereas miR-425-5p was down-regulated by miRNA microarray analysis. qRT-PCR analysis confirmed that the expression of miR-455-3p in TNBC cell lines MDA-MB-231 and MDA-MB-468 was higher than that in HR positive breast cancer cell line MCF-7(*p*<0.01). Functional experiments in vitro showed that miR-455-3p enhanced cell proliferative, invasive and migrational abilities in TNBC cell lines. miRNA targets prediction showed SMAD2, LTBR and etoposide induced 2.4 (EI24) were potential target genes of miR-455-3p, and then it was confirmed by qRT-PCR assay. Dual luciferase reporter assay showed the specific binding of miR-455-3p to 3′ UTR of EI24 in TNBC. Then we found miR-455-3p inhibited the EI24 expression at the levels of mRNA and protein. Through small interfering RNA (siRNA) targeting EI24 gene, there were strengthened capabilities of invasion and migration of TNBC cells, and increased expression of EI24 had the inverse effects. In conclusion, the data suggest that miRNA455-3p promotes invasion and migration by targeting tumor suppressor EI24 and might be a potential prognostic biomarker and therapeutic target in TNBC.

## INTRODUCTION

As proposed at St. Gallen International Expert Consensus meeting [1], breast cancers are divided into 4 subtypes including luminal A-like, luminal B-like, human epidermal receptor-2 (HER-2) positive and triple-negative breast cancer (TNBC) based on immunohistochemistry (IHC) tests for estrogen receptor (ER), progesterone receptor (PR), ki67 together with IHC or *in situ* hybridization (ISH) tests for HER-2 amplification. Representing the most aggressive subtype, TNBC, characterized by the loss of ER, PR, and HER-2 gene expression [2], accounts for 15-20% of all breast cancers, and the majority are basal-like [3]. Compared with other subtypes, TNBC, especially basal-like type, shows the higher risk of metastasis and worse overall prognosis owe to higher histological grade and more aggressive behavior. Extensive research and significant improvements have been achieved in early detection and treatment options for breast cancer. However, due to low-sensitivity responses to hormonal therapy and lack of more specific therapeutic targets, treatment of TNBC remains a challenge [4–6]. Hence, understanding the molecular mechanisms underlying distant metastasis and early relapse of TNBC is pivotal to discover new therapeutic targets and improve prognosis of these patients. Potential pre-treatment values of different molecular markers have been identified, such as androgen receptors (AR), basal cytokeratin, and BRCA genes [7]. Unfortunately, currently none of them can be recognized as a real effective diagnostic or treatment target for TNBC.

MicroRNAs (miRNAs), known as a kind of endogenous small no-coding RNAs which were found in prokaryotic organism and eucaryon, contain 18-22 nucleotides and play important roles in the gene regulation and in a series of pathological and physiological processes such as tumor proliferation, differentiation, progression and apoptosis [8–12]. miRNAs can negatively regulate transcriptional process of target genes or induce the degradation of mRNAs by combining with 3′ untranslated region (3′-UTR), and act as tumor suppressor genes or oncogenes [13]. Approximately one-third of human genes may be regulated by miRNAs, and an individual miRNA can act on hundreds of target genes [14]. Accumulating studies have demonstrated that many miRNAs showed different expression levels and had effects on cellular transformation, carcinogenesis and metastasis in tumors compared with normal tissues [15–18]. Accordingly, the studies on miRNA expression, especially large-scale profiling, have provided evidence that the aberrant expression of miRNA is associated with human breast cancer [19–22]. Yoo et al [23] found the expression of miR-10b increased in metastatic breast cancers, therefore, combining miR-10b-targeted nanotherapy with low-dose doxorubicin elicited durable regressions. Inversely, MiR-145 could inhibit the cellular capabilities of proliferation, infiltration and metastasis in breast cancer. And what is more, some miRNAs are sensitive and specific enough to aid in the detection of breast cancer [24]. However, though a few miRNAs have been reported [25–27], the definite molecular mechanism of miRNAs function has not been well elucidated in TNBC.

In this study, the expression of miRNAs in TNBC versus hormone receptor (HR) positive breast cancer and normal breast tissues was detected by miRNA microarray. We screened out 3 candidate microRNAs showing the most significantly differential expression between TNBC and HR positive breast cancer and miR-455-3p was the most up-regulated miRNAs in TNBC tissues. Then we verified miR-455-3p was up-regulated in TNBC cell lines and TNBC tissues, and found miR-455-3p promoted cell proliferation, invasion and migration through directly targeting etoposide-induced 24 (EI24) *in vitro*.

## RESULTS

### Differential miRNA expression is revealed in TNBC versus HR positive breast cancer specimens by miRNA microarray

**Figure 1:**
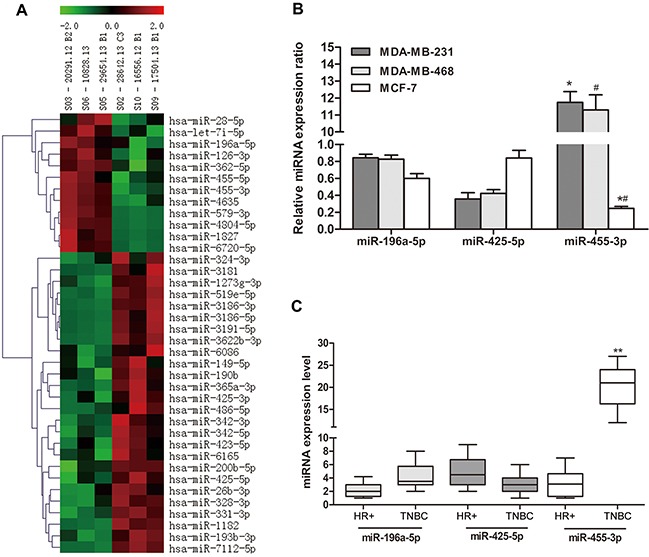
miR-455-3p was up-regulated in TNBC tissues and cell lines **A**. Hierarchical clustering in TNBC (S03, S05, S06) and HR positive breast cancer (S02, S09, S10) on the basis of differentially expressed miRNAs. **B**. Differentially expressed miRNAs in TNBC and HR positive breast cancer including miR-196a-5p, miR-425-5p and miR-455-3p were amplified and detected using qRT-PCR, and miR-455-3p was overexpressed in TNBC cells. **C**. Expression of mRNAs was measured in HR positive breast cancer and TNBC tissues using qRT-PCR.^#^*p<0.01, **p<0.001.

Using the miRNA microarray and data analysis, we screened out 3 candidate microRNAs which showed the most significantly differential expression between TNBC and HR positive breast cancer groups (*p*<0.05), and they were miR-455-3p, miR-425-5p and miR-196a-5p (Figure 1A). miR-455-3p and miR-196a-5p expression were up-regulated, whereas miR-425-5p had a lower expression level in TNBC.

### The expression level of miR-455-3p is up-regulated in TNBC

We quantified the expression levels of these 3 candidate miRNAs and qRT-PCR results demonstrated significant overexpression of miR-455-3p in TNBC cells lines MDA-MB-231 and MDA-MB-468 in comparison with MCF-7 (Figure 1B, *p*<0.05 respectively), however, there was no significant difference of miR-196a-5p and miR-425-5p expression. Further, we investigated the expression of 3 candidate microRNAs in 117 TNBC and 42 HR positive breast cancers. IHC analysis showed that miR-455-3p expression in TNBC tissues was 10-fold more than that in HR positive breast cancer tissues (Figure 1C, *p*<0.001), implying that up-regulation of miR-455-3p might be involved in TNBC development. Therefore miR-455-3p was selected as promising microRNA for further analysis. The association between miR-455-3p expression and clinicopathological parameters was analyzed, as shown in Supplementary Table 1. No significant correlation was identified between miR-455-3p expression and patients’ age, tumor size, histologic grade and lymph node metastasis.

### miR-455-3p improves cell proliferation, invasion and migration abilities in TNBC *in vitro*

**Figure 2:**
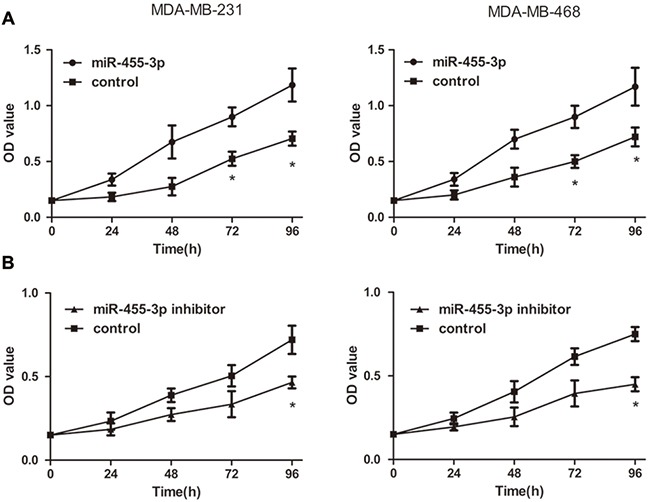
Overexpression of miR-455-3p induced proliferation of TNBC cells *in vitro* Cell viability was determined by MTS assays in MDA-MB-231 and MDA-MB-468 cells transfected with miR-455-3p mimics **(A)** or miR-455-3p inhibitors **(B)** *p<0.05.

**Figure 3:**
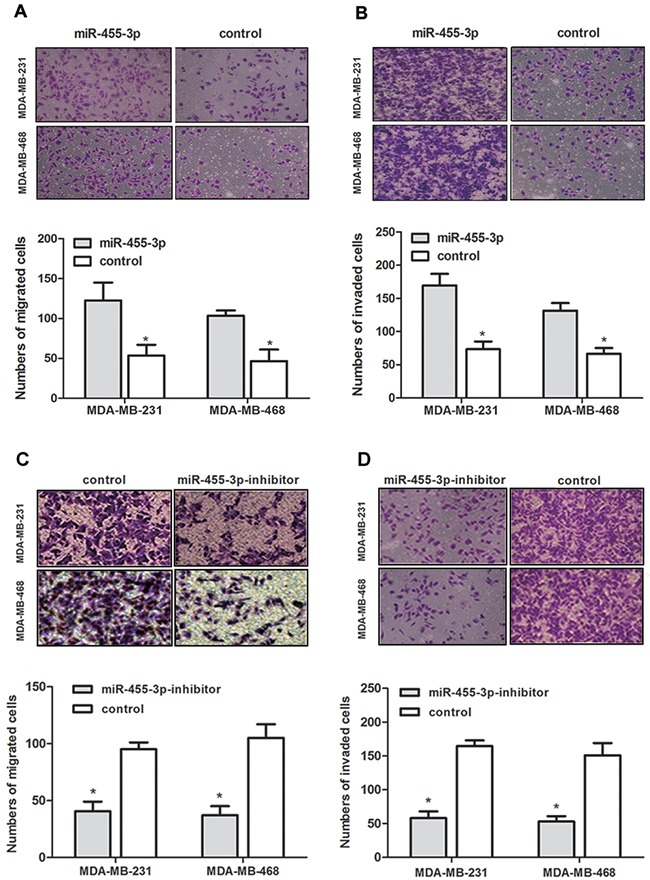
Overexpression of miR-455-3p promoted migration and invasion of TNBC cells *in vitro* Transwell migration **(A, C)** and Matrigel invasion **(B, D)** assays on MDA-MB-231 and MDA-MB-468 cells transfected with miR-455-3p mimics, miR-455-3p inhibitor or negative control showed miR-455-3p promoted cell migration and invasion. *p < 0.05.

To determine the influence of miR-455-3p on biological behavior of TNBC, the TNBC cells were transfected with miR-455-3p mimics, inhibitor and negative control respectively. We examined the proliferation capability of MDA-MB-231 and MDA-MB-468 cells using the MTS proliferation assay. Data demonstrated that overexpressed miR-455-3p promoted TNBC cell proliferation compared with negative control groups at each time point (24 h, 48 h, and 72 h) (Figure 2A, *p*<0.05 for both). In the same way inhibited miR-455-3p showed the opposite effects (Figure 2B, *p*<0.05 for both). The roles of miR-455-3p on the migration and invasion abilities of MDA-MB-231 cells and MDA-MB-468 cells were also investigated. Transwell assays revealed both MDA-MB-231 and MDA-MB-468 cells which have been transfected with miR-455-3p mimics showed improved migration and invasion abilities compared with the negative control groups (Figure 3A, 3B, *p*<0.05 for both). As expected, MDA-MB-231 cells and MDA-MB-468 cells transfected with miR-455-3p inhibitor displayed decreased migration and invasion abilities (Figure 3C, 3D, *p*<0.05 for both). Taken together, miR-455-3p promotes cell proliferation, invasion and migration in TNBC.

### EI24 is the direct target gene of MiR-455-3p

**Figure 4:**
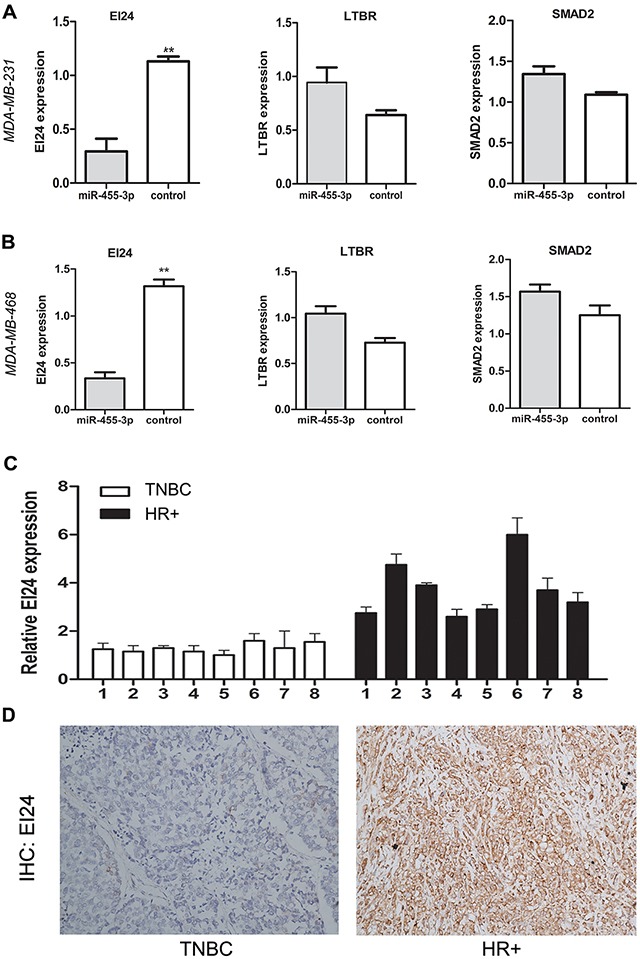
The expression of EI24 was down-regulated in TNBC cells and tissues **A**. The qRT-PCR assay revealed that EI24 mRNA expression was down-regulated significantly in MDA-MB-231 cells transfected with miR-455-3p mimics compared with the cells transfected with miR-control. **B**. Similiar result was obtained in MDA-MB-468 cells. **C**. Representative samples showed that relative EI24 mRNA expression was higher in TNBC tissues than that in HR positive breast cancer using qRT-PCR assays. **D**. Representative IHC stained pictures of EI24 were shown. **p < 0.01.

**Figure 5:**
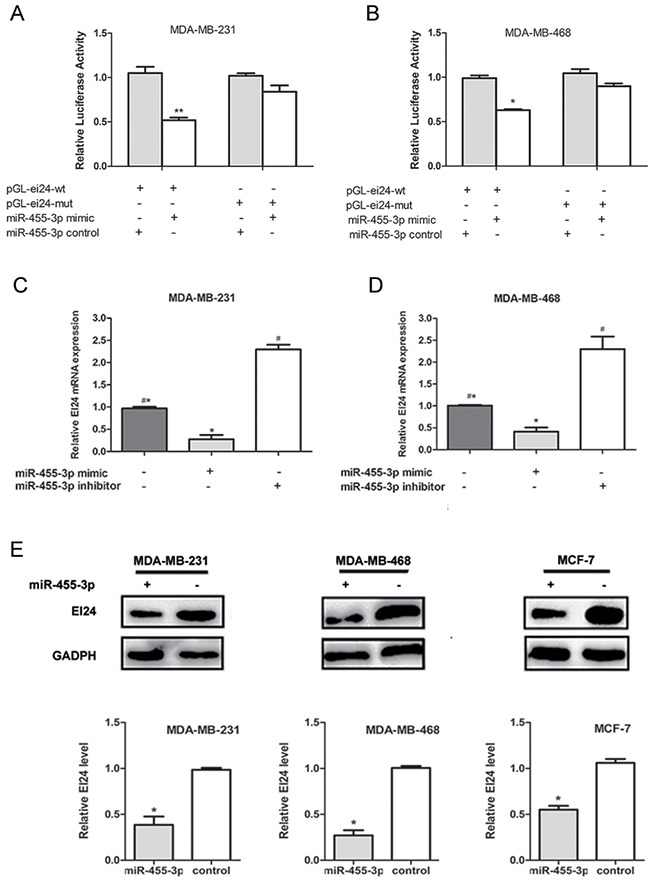
EI24 is a direct target of miR-455-3p **A, B**. Dual-luciferase activity of wild type (WT) EI24 3′UTR reporter was significantly reduced in miR-455-3p mimics compared with the control group in MDA-MB-231 and MDA-MB-468 cells. **C, D**. Expression of EI24 mRNA was detected in TNBC cells transfected with miR-455-3p mimics or inhibitor using qRT-PCR assays. Down-regulation of EI24 protein was observed in miR-455-3p-transfected MDA-MB-231 and MDA-MB-468 cells and HR positive MCF-7 cells, whereas miR-455-3p inhibitor could increase the EI24 expression. **E**. Expression of EI24 protein was detected in TNBC cells transfected with miR-455-3p mimics or inhibitor using Western blot; Decreased EI24 protein expression was observed in the miR-455-3p transfected cells compared with the negative control.^#^* *p*< 0.05.

With the knowledge of miRNAs regulating their target genes via binding to 3′UTR, we searched cancer-related targets of miR-455-3p using online bioinformatics predicting softwares Targetscan, Pictar and Mirbase Family in order to discover the mechanism by which miR-455-3p promotes TNBC cell proliferation and metastasis. We found that EI24, lymphotoxin beta receptor (LTBR) and SMAD family member 2 (SMAD2) were predicted to be the possible target genes. The qRT-PCR assay revealed that EI24 expression was down-regulated in MDA-MB-231 and MDA-MB-468 cells transfected with miR-455-3p mimics (Figure 4A, 4B, *p*<0.01 for both) compared with the cells transfected with miR-control, while the expression of LTBR and SMAD2 were not (Figure 4A, 4B, *p*>0.05 respectively). We also examined the EI24 expression in TNBC and HR positive breast cancer tissue. EI24 mRNA level was lower in TNBC tissues than that in HR positive breast cancer using qRT-PCR assays (Figure 4C). EI24 protein expression detected by IHC showed the similar result. EI24 was located in cytoplasm, and there was a weak or negative EI24 staining in TNBC tissues bearing overexpressed miR-455-3p, whereas there was a diffuse and strong EI24 expression in HR positive breast cancer with low miR-455-3p expression (Figure 4D, *p*<0.05). The dual luciferase reporter assay was carried out to determine whether EI24 was regulated by miR-455-3p directly. 3′-UTR of EI24 containing wild-type (WT) or mutant miR-455-3p target sequences was cloned into the pmirGLO-cad vector. Through co-transfection of miR-455-3p mimics or negative control in MDA-MB-231 and MDA-MB-468 cells, the luciferase activity of the pGL-ei24-wt reporter gene significantly decreased in miR-455-3p mimics group, while the luciferase activity of the pGL-ei24-mut reporter gene was not inhibited. (Figure 5A, 5B; *p*<0.01 and *p*<0.05 respectively). The results indicated that co-transfection of miR-455-3p and EI24 could remarkably repress the luciferase activity in TNBC cells. mRNA level of EI24 was decreased in MDA-MB-468 and MDA-MB-231 cells by transfected with miR-455-3p mimics compared with those in the miR-455-3p inhibitor groups by qRT-PCR (Figure 5C, 5D, *p*<0.001 and *p*=0.001 respectively). Western blot analysis showed that EI24 protein expression was down-regulated as well in miR-455-3p mimics groups of MDA-MB-231, MDA-MB-468 and MCF-7 cells compared with control groups (Figure 5E, *p*<0.05 respectively). These data indicated that miR-455-3p regulated EI24 expression negatively by binding to its 3′-UTR.

### Inhibited EI24 promotes proliferation, migration and invasion of TNBC cells

**Figure 6:**
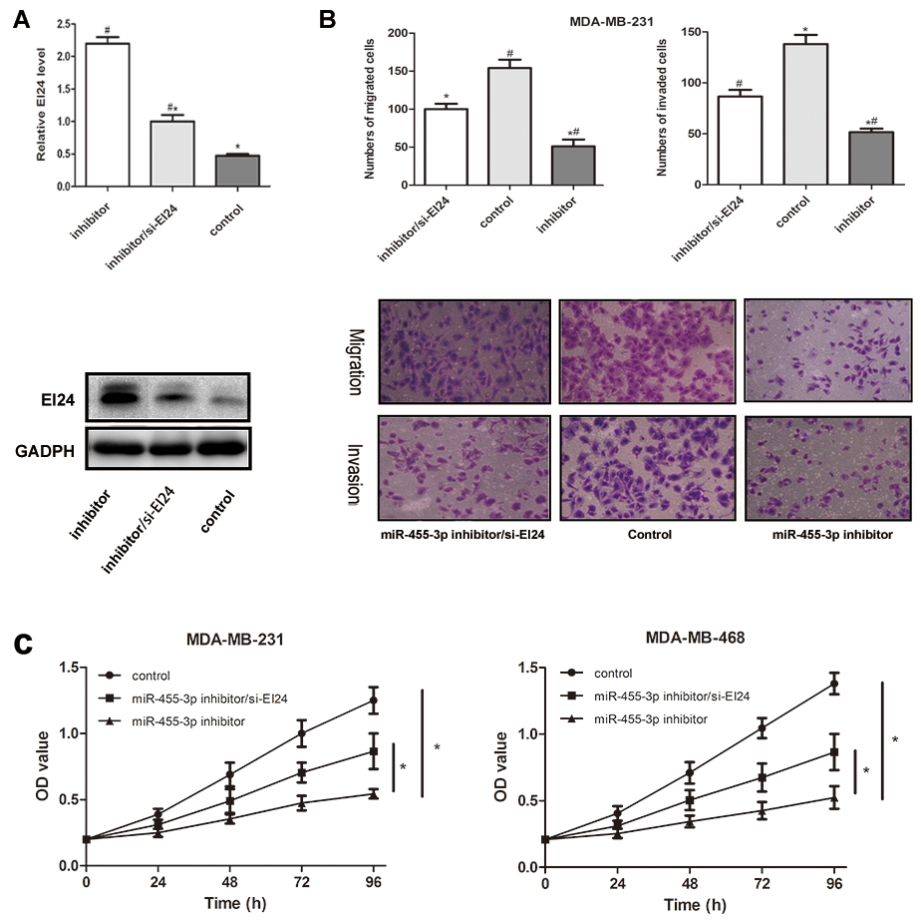
Ectopic expression of EI24 restored the effects of miR-455-3p on cell proliferation, migration and invasion abilities in TNBC cells Cells were respectively co-transfected with miR-455-3p inhibitor (inhibitor) and EI24 siRNA (si-EI24). **A**. Western blot was used to detect the EI24 protein expression. Transfection with miR-455-3p inhibitor promoted the expression of EI24 protein, whereas the si-EI24 reversed the effect of miR-455-3p inhibitor. **B, C**. Migration and invasion capacities by transwell assays, cell proliferation by MTS assays were performed in TNBC cells. It was noted that miR-455-3p inhibitor could inhibited proliferation, migration and invasion abilities of TNBC cells, and transfection of si-EI24 restored the effects of miR-455-3p inhibitor and promoted proliferation, migration and invasion of tumor cells. ^#^* *p* < 0.05.

To investigate whether EI24 was involved in influence of miR-455-3p on the proliferation, migration and invasion of TNBC cells, EI24 siRNA was co-transfected with miR-455-3p inhibitor into cells respectively. Western blot data showed that EI24 siRNA was effective to decrease the expression of EI24 in MDA-MB-231 cells (Figure 6A, p<0.05). Transwell assays indicated that EI24 siRNA partially increased the migration and invasion capabilities of MDA-MB-231 cells compared with the control groups (Figure 6B, *p*<0.05 for both). MTS assays also revealed increased proliferation of miR-455-3p inhibitor-transfected cells (Figure 6C, *p*<0.05 in both MDA-MB-231 and MDA-MB-468 cells). These results suggested that miR-455-3p promoted TNBC proliferation and metastasis by targeting EI24.

## DISCUSSION

TNBC has attracted more attention both clinically and experimentally due to its aggresive biological characteristics and lacking of reliable prognostic markers and effective targeted therapies. Dysregulation of miRNAs is known to be an important mechanism contributing to neoplastic growth and progression. With small sizes miRNAs tend to be more stable than mRNAs [28], therefore, they are good candidates to serve as biomarkers for diagnosis and prognosis evaluation after pathologic biopsies [29, 30]. Recently, the studies have focused on certain single miRNAs to serve as biomarkers and develop effective therapies for TNBC, but mainly based on cell lines [31–33]. In our present study, miRNA array analysis was performed on human TNBC and HR positive breast cancer tissues to screen for triple negative related miRNAs, which can produce more convincing results. We identified miR-455-3p as candidate target and confirmed it was significantly up-regulated in TNBC tissues and cell lines, compared with that in HR positive breast cancer.

In the last decades, there have been few studies reported on miR-455-3p. Guled [34] showed that miR-455-3p was up-regulated in smoker group of malignant mesothelioma patients compared with non-smoker group. Zhang et al [35] revealed that miR-455-3p functioned as an activator for early chondrogenic differentiation by inhibiting the expression of Runt-related transcription factor 2 (Runx2). Moreover, miR-455-3p was found to be linked with acquired temozolomide resistance in glioblastoma multiforme cells [36]. Unfortunately, there is no direct and definite evidence that miR-455-3p is involved in turmorigenesis and development of common malignant tumors. Therefore, to the best of our knowledge, our current study is the first report to confirm its function in tumor progression and identify miR-455-3p as a potential miRNA involved in TNBC development and progression. As a newly identified tumor-promoting miRNA, the finding of miR-455-3p has improved our understanding of the underlying mechanism of TNBC invasion and metastasis and may lead to more efficacious treatment options clinically.

To elucidate the potential mechanism of roles of miR-455-3p in TNBC, three promising genes (EI24, LTBR and SMAD2) were identified to be potential targets of miR-455-3p. EI24 was further proved to be a direct target gene of miR-455-3p. EI24 expression was negatively correlated with miR-455-3p expression, while overexpressed EI24 reversed the effects of miR-455-3p and suppressed proliferation, invasion and migration of TNBC cells. Our findings also demonstrated that EI24 expression was correlated with cell proliferation, invasion and migration. The EI24 expression was dramatically down-regulated while miR-455-3p was up-regulated in TNBC cell and tissues. Moreover, we identified that EI24 has a binding site of miR-455-3p, and observed that overexpression of miR-455-3p decreased the mRNA expression level of EI24. Taken together, these data indicate that miR-455-3p targets EI24 to promote proliferation, invasion and metastasis in TNBC. Similarly, a recent study showed miR-483-3p plays an oncogenic role in esophageal squamous cell carcinoma by targeting EI24 [37].

As the p53-target gene, EI24 is involved in the suppression of cell growth, induction of apoptosis and the activation of autophagy [38, 39]. The human EI24 gene is located on chromosome 11q23, a region frequently displaying loss of heterozygosity (LOH) [40]. Reduced expression of EI24 was found to be associated with the induction of epithelial-to-mesenchymal transition (EMT) and tumor progression by suppressing NF-κB activity [41]. In breast cancer, EI24 is a novel Bcl-2 binding protein which may contribute to apoptosis and low invasiveness by modulating the activity and/or function of Bcl-2 [42]. These results suggest that EI24 may be a valuable biomarker suppressing carcinogenesis and progression, which is consistent with our findings. However, there haven't been studies on effects of EI24 in TNBC.

It has been reported that loss of EI24 contributes to etoposide and gefitinib resistance, suggesting that EI24 status could be used as a prognostic marker for chemotherapy responsiveness [43, 44]. However, in the present study, we did not conduct such studies. In addition, other uncharacterized mechanisms underlying the regulation of EI24 in miR-455-3p-mediated tumor progression remains to be clarified. Thus, much more work is still required to determine its detailed functional mechanisms in TNBC and the potentiality of miR-455-3p as a therapeutic target for TNBC.

In summary, our present study identified 3 miRNAs are dysregulated in TNBC versus HR positive breast cancer, and demonstrates that miR-455-3p is one of the highly expressed microRNAs in TNBC. The data indicate miR-455-3p can promote cell proliferation, migration, invasion abilities by binding to 3′ UTR of EI24 *in vitro*. Our findings suggest miR-455-3p may be involved in TNBC development and progression through miR-455-3p/EI24 axis, which provides potential novel therapeutic target for prevention and treatment of TNBC in the future. However, regarding the development of microRNAs as potential diagnostic or prognostic biomarkers, there is still a long way for us to go.

## MATERIALS AND METHODS

### Patients and tissue samples

117 TNBC and 42 non-TNBC samples (HR positive invasive ducal carcinoma, ER and PR strongly positive) were obtained from patients who underwent radical mastectomy or modified radical mastectomy at the Department of Breast Surgery, Qilu Hospital from 2009 to 2013. As we know, there is about 80% overlap between TNBC and intrinsic basal-like subtype. Some special type of TNBC including such as adenoid cystic carcinoma and metaplastic carcinoma were excluded when we collected patients. None of the patients received chemotherapy or radiotherapy prior to surgery. Two experienced pathologists without knowledge of the clinicopathological data of the patients reviewed H&E slides and immunostaining for all tumors and confirmed the diagnosis. The study was approved by the ethics committee of School of Medicine, Shandong University, China (Approval No. MECSDUMS201). Informed consent was obtained from all patients for the collection of breast cancer specimens in accordance with the guidelines of Qilu Hospital.

### Cell culture

The human TNBC cell lines (MDA-MB-231 and MDA-MB-468) and HR positive cell line MCF-7 were abtained from the American Type Culture Collection ATCC, Manassas, VA, USA). MCF-7 cells were cultured in Dulbecco's modified Eagle's medium (DMEM; Hyclone, Logan, UT, USA) supplemented with 10% fetal bovine serum (FBS; Hyclone, Logan, UT, USA). MDA-MB-468 and MDA-MB-231 cells were maintained in L-15 medium with 10% FBS.

### MicroRNA array and data analysis

Microarray profiling was performed with uParaFlo^TM^ microfluidic array chip (LC.Bio.Tec., Shanghai, China) and raw data were processed and analyzed by Lianchuan Sciences Limited Liability Company. MicroRNA array was conducted on three TNBC, three HR positive breast cancer and three normal breast samples. Differentially expressed miRNAs in TNBC and HR positive breast cancer were identified by cluster analysis as those with a threshold fold change >2, p< 0.05 and FDR<0.1. Then we excluded the miRNAs that were not differentially expressed in TNBC and normal breast tissues. Microarray-based expression findings were validated by qRT-PCR array.

### Target gene predicition of miRNA

Target genes of the candidate miRNA were predicted by using the online softwares including miRBase Target database, TargetScan databases and PicTar databases. The genes which commonly were predicted by at least two prediction tools with a p-value< 0.05 were selected as potential target genes for further analysis.

### Cell transfection and plasmid construction

MDA-MB-231 and MDA-MB-468 cells (1×10^5^ per well) were seeded in 12-well plates. After 24 hours, the cells at 70% confluency were transfected with miR-455-3p mimics, miR-455-3p inhibitor, or the negative controls (GenePharma, Shanghai, China) using lipofectamine 2000 transfection reagent (Invitrogen, USA) according to the manufacturer's protocol. After the transfection, the cells were collected for further assays and the transfection efficiency was detected by qRT-PCR at 24 h after transfection. EI24 coding sequences lacking the 3′UTR were cloned into the pcDNA3.1 vector (Invitrogen, USA) to generate the pcDNA3.1/EI24 expression vector.

### Immunohistochemistry

The formalin-fixed paraffin-embedded slides (4μm) were immunostained with primary antibodies against EI24 (H-20, goat polyclonal antibody, Santa Cruz Biotechnology, Santa Cruz, CA, dilution in 1:200) as described previously [45] and evaluated by LL and XW. Sections incubated with PBS instead of antibodies were used as the negative control. A total of 200 tumor cells chosen randomly in each section were counted and the percentage of positive tumor cells was evaluated. The immunostaining results were categorized as following: negative (<10% cytoplasmic staining); weakly positive (10%-25%); moderately positive (25%-50%); and highly positive (≥50%). Then negative and weak staining was defined as low expression of EI24, while moderate and strong positivity was defined as high expression of EI24.

### qRT-PCR

miRNA extraction was performed with a miRNAeasy FFPE kit (BioTeke Corporation, Beijing, China), which enables the copurification of total RNA, including miRNA, from formalin-fixed paraffin-embedded tissue sections. For cell lines, total RNA was extracted by Trizol (Invitrogen, Carlsbad, CA, USA). After assessment of the quality and concentration of RNA samples using UV-Spectrophotometer (Eppendorf, Germany), the reverse transcription reaction was performed using All-in-One^TM^ miRNA qRT-PCR detection kit (Genecopoeia, USA) according to the manufacturer's instructions. The RNA expression data were analyzed using comparative Ct (2-ΔΔCt) methods [46]. All assays were performed in triplicate. The universal small nuclear RNA U6 (RNU6B) was used as an endogenous control for miRNAs. GAPDH was served as an internal normalization standard for target gene.

### Western blot analysis

Harvested cells were lysed and the total protein was loaded onto 10% gels and transferred to polyvinylidene fluoride (PVDF) membrane. The membranes were blocked for 2 h at room temperature with 5% non-fat milk in TBST (Tris-buffered saline containing 0.1% Tween-20). Then the membranes were incubated overnight at 4°C with anti-EI24 or anti-GADPH primary antibodies and subsequently washed three times in TBST for 5 min. After incubation with horseradish peroxidase-conjugated secondary antibodies for 2 h at room temperature, they were washed with TBST. Lastly, the protein bands on membranes were examined and recorded by an enhanced chemiluminescence system (ECL). Relative protein expression was normalized to GAPDH.

### Luciferase reporter assay

Luciferase reporter assays were carried out in both MDA-MB-231 and MDA-MB-468 cell lines. 1×10^5^ cells were seeded in 12-well plates 24 h before transfection. miR-145 precursor or miR-negative control and pmirGLO-3′UTR vector were co-transfected into cells using lipofectamine 2000 (Invitrogen, USA). Luciferase activities were detected with a Dual-Luciferase Reporter Assay Kit (Promega, USA) according to the manufacturer's protocol. The absorbance of Firefly and Renilla luciferase activity was detected by a Microplate Luminometer (Berthold Technologies). Each experiment was performed in triplicate.

### Cell proliferation assay

The MTS assay was performed to provide a preliminary assessment of proliferation affected by miR-455-3p using the MTS solution cell proliferation assay kit (Promega Corporation, WI, USA). The MTS assay was performed at 24 h, 48 h, 72 h respectively. The absorbance at 490 nm was read using an automatic plate reader (BIO-RAD, USA), and the final absorption (A) values were the values in each group minus the background absorbance values. The cell number was expressed as mean and standard deviation of triplicate measurements.

### Cell migration and invasion assays

The migration assay was conducted with transwell plates with 8μm pores (Corning Incorporated, Corning, NY, USA). Cell invasion assays were performed using invasion chambers (Corning Incorporated, Corning, NY, USA) pre-coated with Matrigel. Cells (2×10^5^) were resuspended in serum-free medium and seeded into the upper chamber. Culture medium containing 20% fetal bovine serum (FBS) was added to the lower chamber as the chemoattractant. The cells were incubated in a humidified incubator at 37°C for 24 h (migration assay) or 36 h (invasion assay). Non-invading cells in the upper chambers were removed with cotton swabs. The cells attached to the lower surface were fixed and stained. The number of cells which attached to the lower surface was counted in five random fields under a microscope (×200).

### Statistical analysis

All data analyses were performed using Statistical Package for the Social Sciences version 19.0 software (SPSS, Chicago, IL, USA). Data are present as mean ± standard deviation. The means in different groups were compared by two-tailed, unpaired Student's test. And the statistical significance was determined by Mann-Whitney test or one-way analysis of variance (ANOVA). Statistical significance was accepted at *p*<0.05.

## SUPPLEMENTARY TABLE


