# Supplementary material for: MicroRNA-455-3p promotes invasion and migration in triple negative breast cancer by targeting tumor suppressor EI24
Source: Oncotarget. 2016 Dec 27;8(12):19455–66. doi: 10.18632/oncotarget.14307 (PMC5386697; doi:10.18632/oncotarget.14307)
Supplement: Supplementary file 1 [file oncotarget-08-19455-s001.pdf]

MicroRNA-455-3p promotes invasion and migration in triple negative breast cancer by targeting tumor suppressor EI24

SUPPLEMENTARY TABLE

Supplementary Table 1: Correlation of clinicopathological variables with miR-455-3p expression in 117 TNBC specimens

Variables	No.	MiR-455-3p		*p*
		Low	High	
**Age**				**0.532**
≤35y	11	4	7	
>35y	106	49	57	
**Size**				**0.792**
≤2cm	38	16	22	
2-5cm	76	36	40	
>5cm	3	1	2	
**Grade**				**0.704**
I	3	1	2	
II	30	12	18	
III	84	40	44	
**Lymphnode**				**0.734**
0	62	29	33	
+	55	24	31	
